# Harnessing the Power of Community Engagement for Population Health

**DOI:** 10.5888/pcd22.250189

**Published:** 2025-06-05

**Authors:** Tabia Henry Akintobi, Robert E. Bailey, J. Lloyd Michener

**Affiliations:** 1 Prevention Research Center, Morehouse School of Medicine, Atlanta, Georgia; 2 Centers for Disease Control and Prevention, Atlanta, Georgia; 3 Department of Family Medicine and Community Health, Duke School of Medicine, Durham, North Carolina

Community engagement has a long history in public health and in the prevention of chronic disease (1,2). In 1997, the Centers for Disease Control and Prevention (CDC) and the Agency for Toxic Substances and Disease Registry (ATSDR), published the first edition of the *Principles of Community Engagement*, noting that community involvement and collaboration had become the foundation of public health action (3). In 2011, a second edition was developed in partnership with the Clinical and Translational Science Awards (CTSA) Program of the National Institutes of Health (NIH). It added the concept of engagement as a continuum from outreach to shared leadership, examples from the field, and implementation and evaluation guidance (4). A third edition was published in 2025 as a collaboration among the CTSA Program, NIH, ATSDR, and CDC, with some 165 authors spanning community organizations, academia, and federal agencies (5).

Definitions of community and community engagement and their key elements have evolved. The third edition of the *Principles of Community Engagement* notes that communities can be thought of as a group of people with diverse characteristics who are linked by social ties, shared common perspectives and identity, and engagement in joint action. A single person may belong to many communities (5). Community engagement is the process of building sustainable relationships through trust and collaboration that strengthens community well-being. The process should be enduring, equitable, and culturally sensitive to all participants, with a shared goal of addressing the concerns of the community. The third edition adds the principle of trustworthiness as a fundamental element in sustaining community engagement and advancing health equity (6).

The National Academy of Medicine launched a major effort on meaningful community engagement in health with a primary report released in 2022 (7). An organizing committee of community leaders, researchers, and policy advisors was charged with compiling and assessing community-engaged and evidence-based tools that could be used to ensure that engagement is meaningful to communities. The organizing committee realized the need for a new conceptual model that illustrates the dynamic relationship between community engagement and improved health and health care outcomes. The new model highlights the centrality of community engagement; the core principles required for meaningful, successful, and sustained engagement; and how meaningful engagement leads to strengthened partnerships, expanded knowledge, improved health, and transformed systems that provide everyone with the opportunity for health. A linked set of stories and measurement instruments are provided, mapped to domains of the conceptual model (Figure).

**Figure:**
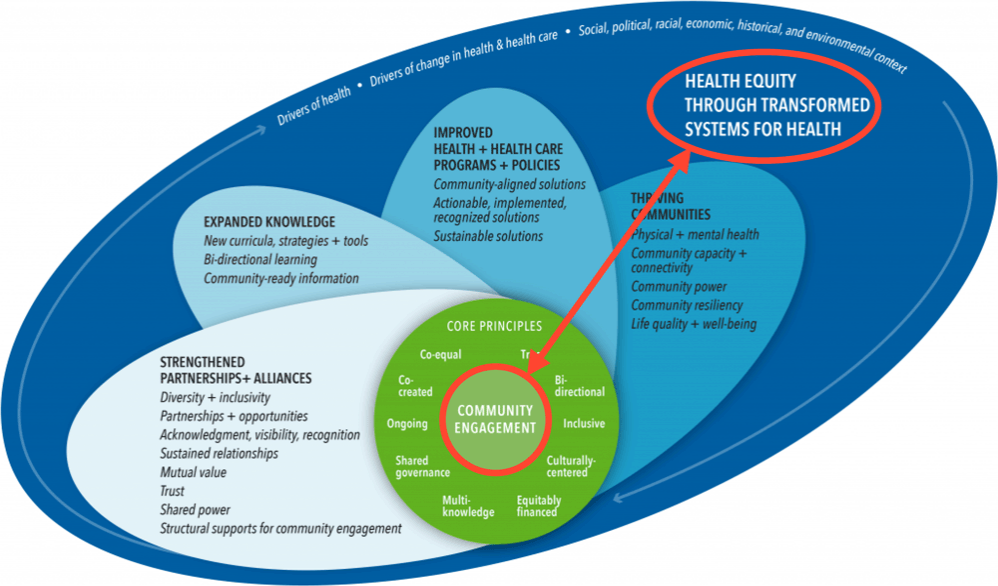
A conceptual model to advance health equity through transformed systems for health. Elements in red were added by the authors. Adapted with permission from the National Academy of Medicine. The model is available online at https://nam.edu/product/achieving-health-equity-and-systems-transformation-through-community-engagement-a-conceptual-model.

Interest in community engagement in public health continues to grow. A 2021 bibliographic mapping of the topic noted more than 1,100 publications; the number of publications increased sharply from 1980 to 2020 and half of the published reports were from the US (8). These models and their applications to public health were discussed in a recent editorial in the *American Journal of Public Health* (9). Other recent reports detail multisectoral community-engaged research and practice programs and models addressing underlying determinants (10,11). A toolkit and discussion guide on trustworthiness was developed by the Center for Health Justice of the Association of American Medical College with extensive community guidance, reflecting the need for enhanced attention to trust and trustworthiness (12).

Together, these reports heighten the emphasis on the role of community engagement in public health. Recent publications in *Preventing Chronic Disease* (PCD) also highlight the role of community engagement, including examples of how and where engagement has been supported and effective so that public health efforts to prevent chronic disease are trustworthy, effective, and sustained.

## PCD Collection on Community Engagement and Population Health: From Practice to Evaluation

This collection of 9 PCD articles focuses on community engagement in public health, from practice to evaluation. Historically, community engagement has been commonly found in formative research activities, informing development of communication strategies, messages, and tools, as well as prioritizing issues and policy solutions. Brewer et al describe 2 boot camps to develop locally relevant materials on the risk of human papillomavirus among vaccine-eligible children, adolescents, and young adults, noting differences in both messages and presentation methods developed by various communities and reinforcing the value of local community input (13). Olson et al describe identification of hot spots for female breast cancer and lung cancer and a statewide effort, led by the Advancing a Healthier Wisconsin Endowment, to engage people from different backgrounds and communities about the causes and potential strategies for addressing disparities (14). Listening sessions noted a broad range of contributors to cancer disparities, areas with shared knowledge, and areas in which further discussion and education (both of the public and scientific community) were likely needed. Researchers were noted to have general knowledge of the role of social context in cancer disparities, while community participants had extensive knowledge of the complex community-specific interplay between social relationships, social conditions, and policy.

Communities have been increasingly involved in mapping strategies to prioritize needs and interventions. Payán et al describe application of a mapping component for a multilevel church-based intervention that used community-based participatory research to prevent obesity among church members in South Los Angeles (15). Multiple dimensions of food insecurity were documented, varying across neighborhoods, as was the need for additional work on translating mapped data to policy and local environmental interventions. Thompson et al used community-engaged concept mapping to generate consensus on priorities for care, research, and cancer control in Kentucky (16). Adult community members and staff members of statewide and community-based partner organizations were recruited to participate in a video-conferencing concept mapping process. These researchers found a high rate of congruence among topics and potential strategies. Keller et al describe a community–academic partnership between residents of Milwaukee’s Near West Side and Marquette University to generate, sort, and rate maps of clusters of concepts of a healthy community, showing how new tools can bring together ideas that have broad support and become the foundation for strength-based solutions aligned with partner priorities (17).

Other articles in this collection reinforce the idea that community engagement needs to be a deliberate effort with tangible results. Carnahan et al describe how the Illinois Department of Public Health adopted a robust community- and legislative-engaged approach that reflected the voices of people affected by cancer and the diverse needs and assets in the state (18). DeBruyn et al describe the design, implementation, and evaluation of community-defined strategies to address type 2 diabetes across 17 tribes and tribal communities by focusing on traditional foods, physical activity, and social support (19). Using a mixed-method evaluation, they found an increase in targeted activities, challenges in evaluation when multiple groups work together, and the need for sustained community infrastructure. Elliott et al describe an extensive community health program with some 25 community partners and Duquesne University, coordinated by the Allegheny County Health Department (20). The program included community-based screening with counseling by a pharmacist and referral to additional clinicians and/or community service providers. Qualitative evaluation found that the program provided needed services but was hampered by challenges in follow-up, inadequate community resources, and need for sustained funding.

Finally, Kepper et al describe a more than 2-year process by multiple community-based health organizations in St. Louis metropolitan areas to address, test, and evaluate interventions to optimize health for all, particularly those living in a federally designated Promise Zone (21). This complex project evolved throughout the COVID-19 pandemic, accelerated activities to online platforms, expanded internet accessibility for people with poor connectivity, and demonstrated the resilience of engaged community groups.

## Where We Go From Here

Community engagement is central to public health and chronic disease prevention. The articles in this collection showcase examples of engagement with local and state communities. They use data and community wisdom to inform decision-making, adaptation, and implementation; highlight the resilience of communities; and document challenges in program implementation, follow-up, and sustainment. These articles and their examples, together with other national work on community engagement in health, yield several recommendations for public health practitioners:

- Go to communities to learn their perspectives, strengths, values, and priorities. This is central to intentional relationship-building, reflecting the value of community members and groups as central to planning, implementing, evaluating, and sustaining programs and research that matters. Despite funding challenges, this practice is central to trust, must be built over time, and should not be rooted in the acquisition or administration of a grant.
- Amplify and credit the community wisdom central to ideation, process, implementation, evaluation, and recommendations, including investigator status, compensation, co-authorship, co-presentation, and co-branding.
- Address institutional and organizational barriers to and needed investments in community-engaged prevention practice and research. Barriers may reflect deep-seated administrative structures that threaten efficiency and trust even among the most well-meaning, mission-aligned partners.
- Partner with health care, social service, business, faith, and nongovernment organizations to address social and political factors associated with health and health care. These potential partners are often underused, despite their services and ability to influence health priorities such as housing, workforce development, food access, and primary health care.
- Support community-led projects and infrastructure central to sustained success. Mechanisms that position communities as senior or principal investigators of prevention programs and research are essential to powering (not empowering) their leadership and sustaining their programs. This value and practice must be bolstered by partnerships and resources for rigorous and robust data collection and analysis to demonstrate impact and outcomes.
- Address and eradicate rampant health misinformation and disinformation by reimagining public health communication, in partnership with community influencers, resulting in messages that are not only accurate but attend to social motivation, lived experiences, and trusted sources across the spectrum of mass and social media communication.
- Advocate for community-led public health improvement. Community-informed data systems, metrics, and networks should not only drive responsive research, practice, and clinical care but also be the change that dismantles systemic and structural barriers to health through local, regional, and national policy.
- Practice the values of listening to understand, cultural humility to translate, and trustworthiness to build and trust.
- Respect community strengths and avoid the idea that communities lack resources and need the preconceived solutions of outside groups to solve problems within the community.

Together these practices will expand community-engaged public health research, practice, and action and build community trust. Community-engaged prevention of chronic disease is realized through integrated efforts in education, research, clinical care, and service, in collaboration with partners committed to improving health outcomes and addressing the root causes of health inequities. These root causes are embedded in systems, conditions, and contexts that support or prohibit optimal health. Public health practitioners who embody these values engage in early and sustained community assessments to deepen understanding of local assets, needs, histories, and power dynamics.

Community engagement has been identified as a core attribute of public health for 4 decades and is a necessity for building trust in the decades to come. Preventing chronic disease occurs through the active, meaningful engagement of communities, who co-design, implement, and evaluate the programs or research they prioritize and which they decide to lead, support, and sustain.

## References

[1] Hanson P . Citizen involvement in community health promotion: a role application of CDC’s PATCH model. *Int Q Community Health Educ.* 1988;9(3):177–186. 10.2190/FMWL-59TW-T3CL-VJ16 20841203

[2] Puska P , Salonen JT , Tuomilehto J , Nissinen A , Kottke TE . Evaluating community-based preventive cardiovascular programs: problems and experiences from the North Karelia project. *J Community Health.* 1983;9(1):49–64. 10.1007/BF01318933 6678258

[3] Centers for Disease Control and Prevention. *Principles of Community Engagement.* 1st ed. CDC/ATSDR Committee on Community Engagement; 1997.

[4] US Department of Health and Human Services. *Principles of Community Engagement.* 2nd ed. US Government Printing Office; 2011.

[5] Centers for Disease Control and Prevention, Agency for Toxic Substances and Disease Registry. *Principles of Community Engagement.* 3rd ed. Centers for Disease Control and Prevention, Agency for Toxic Substances and Disease Registry; 2025.

[6] Association of American Medical Colleges Center for Health Justice. The Centers for Disease Control and Prevention adds a new principle of community engagement. January 21, 2025. Accessed May 8, 2025. https://www.aamchealthjustice.org/news/news/centers-disease-control-and-prevention-adds-new-principle-community-engagement

[7] Aguilar-Gaxiola S , Ahmed SM , Anise A , Azzahir A , Baker KE , Cupito A , . Assessing meaningful community engagement: a conceptual model to advance health equity through transformed systems for health: organizing committee for assessing meaningful community engagement in health & health care programs & policies. *NAM Perspect.* 2022;2022(2). 10.31478/202202c 35891775 PMC9303007

[8] Yuan M , Lin H , Wu H , Yu M , Tu J , Lü Y . Community engagement in public health: a bibliometric mapping of global research. *Arch Public Health.* 2021;79(1):6. 10.1186/s13690-021-00525-3 33436063 PMC7801880

[9] Michener JL , Williams A , Oto-Kent D , Aguilar-Gaxiola SA . Community engagement: a foundation for health equity and resilience. *Am J Public Health.* 2025;115(S2):e1–e6. 10.2105/AJPH.2025.308029 PMC12199671 40561398

[10] Adsul P , Sanchez-Youngman S , Dickson E , Jacquez B , Kuhlemeier A , Muhammad M , . Assessing the context within academic health institutions toward improving equity-based, community and patient-engaged research. *J Clin Transl Sci.* 2024;9(1):e6. 10.1017/cts.2024.675 39830606 PMC11736299

[11] Key KD , Furr-Holden D , Lewis EY , Cunningham R , Zimmerman MA , Johnson-Lawrence V , . The continuum of community engagement in research: a roadmap for understanding and assessing progress. *Prog Community Health Partnersh.* 2019;13(4):427–434. 10.1353/cpr.2019.0064 31866597

[12] Association of American Medical Colleges Center for Health Justice. The principles of trustworthiness. 2025. Accessed May 18, 2025. https://www.aamchealthjustice.org/our-work/trustworthiness/trustworthiness-toolkit#principles

[13] Brewer SE , Nederveld A , Simpson M . Engaging communities in preventing human papillomavirus–related cancers: two boot camp translations, Colorado, 2017–2018. *Prev Chronic Dis.* 2020;17:E02. 10.5888/pcd17.190250 31895672 PMC6977779

[14] Olson J , Cawthra T , Beyer K , Frazer D , Ignace L , Maurana C , . Community and research perspectives on cancer disparities in Wisconsin. *Prev Chronic Dis.* 2020;17:E122. 10.5888/pcd17.200183 33034557 PMC7553208

[15] Payán DD , Derose KP , Flórez KR , Branch CA , Williams MV . The food environment in 3 neighborhoods in South Los Angeles, California: access, availability, quality, and marketing practices. *Prev Chronic Dis.* 2020;17:E61. 10.5888/pcd17.200028 32678063 PMC7380293

[16] Thompson JR , Burus T , McAfee C , Stroebel C , Brown M , Francis K , . A community-engaged, mixed-methods approach to prioritizing needs in a statewide assessment of community cancer needs. *Prev Chronic Dis.* 2024;21:E103. 10.5888/pcd21.240183 39724002 PMC11675799

[17] Keller AO , St. Arnold Bell L , Haglund K . Engaging a community–academic partnership to implement community-driven solutions. *Prev Chronic Dis.* 2025;22:240334. 10.5888/pcd22.240334 PMC12151494 40471851

[18] Carnahan LR , Hallock C , Soto B , Kasebier L , Dracos E , Martinez E , . Creating and implementing a community engagement strategy for the 2022–2027 Illinois Comprehensive Cancer Control Plan through an academic-state public health department partnership. *Prev Chronic Dis.* 2023;20:E69. 10.5888/pcd20.220422 37562068 PMC10431926

[19] DeBruyn L , Fullerton L , Satterfield D , Frank M . Integrating culture and history to promote health and help prevent type 2 diabetes in American Indian/Alaska Native Communities: traditional foods have become a way to talk about health. *Prev Chronic Dis.* 2020;17:E12. 10.5888/pcd17.190213 32027813 PMC7021459

[20] Elliott JP , Christian SN , Doong K , Hardy HE , Mendez DD , Gary-Webb TL . Pharmacist involvement in addressing public health priorities and community needs: the Allegheny County Racial and Ethnic Approaches to Community Health (REACH) project. *Prev Chronic Dis.* 2021;18:E07. 10.5888/pcd18.200490 33507859 PMC7845549

[21] Kepper M , Stamatakis KA , Mudd N , Deitch A , Terhaar A , Liu J , . A communitywide collaboration to increase enrollment, retention, and success in evidence-based lifestyle-change programs in racial and ethnic minority populations. *Prev Chronic Dis.* 2023;20:E67. 10.5888/pcd20.220352 37535902 PMC10431923

